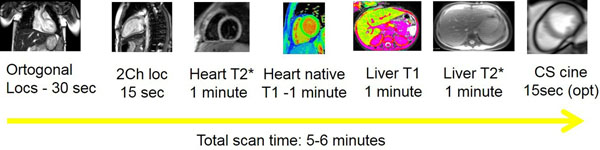# Use of an accelerated protocol for rapid analysis of iron overload in the heart and liver: the All Iron Detected (AID) Multicenter Study

**DOI:** 10.1186/1532-429X-17-S1-O62

**Published:** 2015-02-03

**Authors:** Juliano L Fernandes, Maria Helena A Siqueira, Karina T Nobrega de Oliveira, Luiz Francisco Avila, Ilan Gottlieb, Marly U Lopes, Andre M Fernandes, Ralph Strecker, Andreas Greiser

**Affiliations:** 1 Jose Michel Kalaf Research Institute, Campinas, Brazil; 2 Radiologia Clinica de Campinas, Campinas, Brazil; 3 Santa Joana Diagnostico, Recife, Brazil; 4 Hospital Sirio Libanes, Sao Paulo, Brazil; 5 CDPI, Rio de Janeiro, Brazil; 6 DASA, Sao Paulo, Brazil; 7 Hospital Ana Nery, Salvador, Brazil; 8 Siemens Ltda, Sao Paulo, Brazil; 9 Hospital Mater Dei, Belo Horizonte, Brazil; 10 Siemens AG, Erlangen, Germany

## Background

Magnetic resonance imaging has become an essential tool in the management of patients with iron overload. Most of these patients are located in under-developed regions of the world where access to scanners is limited. Effective use of resources is therefore mandatory especially in very densely populated areas. We sought to assess if an accelerated protocol under ten minutes for iron overload assessment in the heart and liver using automated sequences and analysis would be feasible and accurate in a multicenter study.

## Methods

Seven centers participated in the study enrolling 179 patients in ten-minute slots. All patients underwent a similar protocol using different 1.5T scanners from a single vendor (Siemens AG), consisting of orthogonal localizers, two-chamber localizer, single-slice short-axis prototype T1 MOLLI and T2* multiecho acquisition of the heart and single-slice transaxial slice of the liver with T1 and T2* sequences. Automated maps for both T2* and T1 were generated by an in-line processing prototype software. T2* results were also obtained by manual calculation using a ROI-based fit and truncation. In a subgroup of sixteen patients, left-ventricle function was also assessed using a sparse sampling and iterative reconstruction cine technique with a repeat scan using traditional T2* sequences plus cine-SSFP imaging. Endpoints of the study included the time required for each exam, the accuracy of the automatically calculated T2* values compared to the manual analysis as well as factors associated with duration of the exam and successful completion of the protocol.

## Results

The mean age of participants was 27.5±20.1 years (range 2 to 91 years) with 51% females and 44% children/adolescents. The median scan time was 5.2 minutes (IQR 4 to 7 minutes). For the heart, Bland-Altman plots demonstrated a mean difference of -1.2ms (95%CI -1.7 to -0.8ms) for the automated maps compared to the manual analysis. In the liver, this difference was -0.18ms (95%CI -0.30 to -0.06ms). In only 1.1% of the patients, the heart T2* values could not be calculated due to motion; all liver T2* values were obtained. Age, sex, etiology and hemoglobin levels did not affect the total exam time while significant differences were observed among the centers (P<0.001). In the subgroup of sixteen patients with repeat scans including cine imaging, there were significant differences in total time of scan (7.5±1.8min vs 10.2±2.5min, P=0.001).

## Conclusions

Use of an accelerated protocol for acquisition and calculation of iron overload in the liver and heart is feasible and results in successful completion of the study in the majority of patients with similar values compared to manual analysis.

## Funding

N/A.

**Figure 1:**